# The Potential of *Moringa oleifera* to Ameliorate HAART-Induced Pathophysiological Complications

**DOI:** 10.3390/cells11192981

**Published:** 2022-09-24

**Authors:** Siqiniseko S. Ndlovu, Terisha Ghazi, Anil A. Chuturgoon

**Affiliations:** Discipline of Medical Biochemistry, School of Laboratory Medicine and Medical Sciences, University of KwaZulu-Natal, Durban 4041, South Africa

**Keywords:** HIV, HAART, pathophysiology, metabolic syndrome, *Moringa oliefera*

## Abstract

Highly active antiretroviral therapy (HAART) comprises a combination of two or three antiretroviral (ARV) drugs that are administered together in a single tablet. These drugs target different steps within the human immunodeficiency virus (HIV) life cycle, providing either a synergistic or additive antiviral effect; this enhances the efficiency in which viral replication is suppressed. HIV cannot be completely eliminated, making HAART a lifetime treatment. With long-term HAART usage, an increasing number of patients experience a broadening array of complications, and this significantly affects their quality of life, despite cautious use. The mechanism through which ARV drugs induce toxicity is associated with metabolic complications such as mitochondrial dysfunction, oxidative stress, and inflammation. To address this, it is necessary to improve ARV drug formulation without compromising its efficacy; alternatively, safe supplementary medicine may be a suitable solution. The medicinal plant *Moringa oleifera* (MO) is considered one of the most important sources of novel nutritionally and pharmacologically active compounds that have been shown to prevent and treat various diseases. MO leaves are rich in polyphenols, vitamins, minerals, and tannins; studies have confirmed the therapeutic properties of MO. MO leaves provide powerful antioxidants, scavenge free radicals, promote carbohydrate metabolism, and repair DNA. MO also induces anti-inflammatory, hepatoprotective, anti-proliferative, and anti-mutagenic effects. Therefore, MO can be a source of affordable and safe supplement therapy for HAART-induced toxicity. This review highlights the potential of MO leaves to protect against HAART-induced toxicity in HIV patients.

## 1. Introduction

The World Health Organization (WHO) reported that there are 38 million people currently living with human immunodeficiency virus (HIV) globally, and the majority of these individuals are in South Africa (SA) [1]. SA has an HIV infection prevalence of 19% and carries the largest disease burden worldwide [2,3]. HIV is suppressed through the effective use of antiretroviral (ARV) drugs [4]. Over the past years, ARV formulations have been improved, and when combined with two or three ARVs from different ARV drug classes, make a highly active antiretroviral therapy (HAART), also known as antiretroviral therapy (ART).

The implementation of HAART prolongs the life expectancy in HIV-infected individuals, and HAART has led to a significant decline in morbidity and mortality among HIV-infected patients [5,6]. Despite its high effectiveness to suppress HIV viral replication, HAART cannot completely eliminate the virus because of the presence of multiple T-cell reservoirs [7] and, for this reason, HIV-infected patients need to be on HAART throughout their lifetime in order to keep their viral load under 50 copies/mL [8,9]. As a result of HAART being a life-long treatment, adverse outcomes associated with this long-term therapy have been emerging.

HAART has evolved with the intention to make it less toxic, while optimizing its function; however, it is not void of toxicity. The ART regimen of tenofovir disoproxil fumarate (TDF), lamivudine (3TC), emtricitabine (FTC), dolutegravir (DTG), and efavirenz (EFV), in the long-term, has been associated with the development of pathophysiological complications, referred to as metabolic syndrome (MetS) [10,11,12]. MetS is a combination of metabolic disorders that include hypertension, hyperglycemia, changes in fat distribution, increased cholesterol low-density lipoprotein (LDL) and triglycerides, and reduced levels of cholesterol high-density lipoprotein (HDL), which may lead to cardiovascular diseases (CVDs) such as heart disease, stroke, and diabetes [13,14,15,16,17,18,19]. It is therefore of paramount importance that constant approaches for the improvement of ART treatment are made a clinical and pharmaceutical priority. Alternatively, the use of supplementary medicine such as medicinal/herbal plants may provide a possible solution.

Medicinal plants and phytomedicines are believed to have benefits over conventional drugs and are regaining interest in current research. * Moringa oleifera* (MO) is a medicinal plant that has been identified for its nutritious and therapeutic benefits. All parts of this plant have a notable range of functional and nutraceutical properties [20]. Several studies have demonstrated beneficial effects in humans [21,22,23,24]. MO is a rich source of several phytochemicals, such as phenols, flavonoids, vitamins, minerals, quercetin, and kaempferol. In addition, it also contains carotenoids, phenolic acids, alkaloids, glucosinolates and isothiocyanates [25].

MO leaves provide powerful antioxidants [26,27], free radical scavenging [28], anti-inflammation, anti-eNOS expression [29,30], anti-mutagenic, anti-proliferative, anticancer [31,32], hepatoprotective [33], carbohydrate metabolism promoter [34], and repairs DNA [31]; moreover, MO leaves are a rich source of essential amino acids [35,36], thus validating the therapeutic claims.

In this review, we outline the toxic effects of HAART/ART and propose MO as a potential supplementary medicine to ameliorate these toxicities.

## 2. HAART-Induced Mitochondrial Toxicity and Oxidative Stress

The long-term use of ARV drugs contributes to long-term complications in HIV-infected persons. Mitochondrial dysfunction and oxidative stress are highlighted as metabolic pathways through which ARV drugs induce MetS [37,38,39,40]. Nucleoside reverse transcriptase inhibitors (NRTIs), which are a cornerstone of HAART regimens, non-nucleoside reverse transcriptase inhibitors (NNRTIs), protease inhibitors, and integrase strand transfer inhibitors (PIs/INSTIs) have been noticeably associated with many adverse effects related to mitochondrial toxicity and oxidative stress [41,42,43,44].

**Figure 1:**
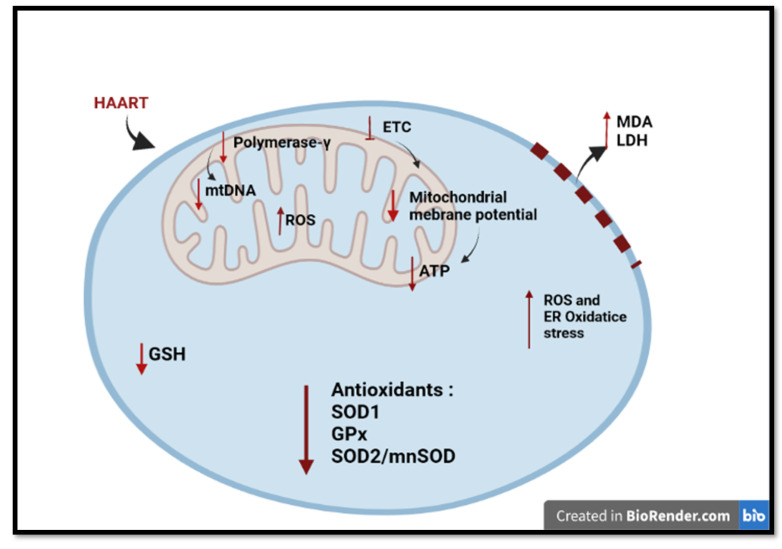
An overview of long-term HAART mitochondrial toxicity and oxidative stress in HIV-positive individuals. HAART interferes with the synthesis of polymerase-γ, reducing the mtDNA. This therapy impairs ETC, increasing ROS production, depolarizing the mitochondrial membrane, and compromising the ATP synthesis. HAART also depletes GSH and other cellular antioxidants, propagating oxidative stress in the cell. Created with BioRender.com (access date: 2 June 2022).

The effects of HAART were observed in the mitochondria (Figure 1). NRTIs found in HAART inhibit the activity of DNA polymerase-γ, an enzyme responsible for the replication and maintenance of mitochondrial DNA (mtDNA), thus compromising mitochondrial integrity and function [40,45]. The triphosphate (active) forms of NRTI are potential substrates for the polymerase-γ and can provoke the termination of the DNA chain during mtDNA replication [46]. The mtDNA depletion also leads to an impaired synthesis of mtDNA encoded respiratory chain polypeptides, which can partially block the flow of electrons in the respiratory chain. As a result, they accumulate in complex I and III, where they react with oxygen to form the superoxide anion radical [47]. These effects have been described with selected NRTIs [48,49,50].

The NRTIs impairs oxidative phosphorylation (OXPHOS) proteins and increases oxidative stress in the mitochondria. This leads to damage of mitochondrial proteins and lipids further impairing mitochondrial function [51,52] Mitochondrial dysfunction by NRTIs is also manifested by depolarization of the mitochondrial membrane and increased reactive oxygen species (ROS) generation [49]. NRTI further interferes with the synthesis of essential proteins of the mitochondrial electron transport chain (ETC), causing alterations in nucleotide phosphorylation, directly interfering with mitochondrial respiration and reduce ATP production [53,54]. NRTIs also impair respiration and ATP synthesis, by preventing ATP/ADP translocation [54].

Not all NRTIs exert the same degree of polymerase-γ inhibition; however, they have the capacity to induce mitochondrial toxicity. In vitro studies have demonstrated that 3TC inhibits polymerase-γ, although its affinity for polymerase-γ is not as strong as the previously discontinued ARVs [55,56]. Samuels, Bayerri [57] reported that mtDNA deletion mutation was detectable significantly more commonly in the urine of TDF exposed study participants as compared to unexposed individuals.

FTC effects on the mitochondria include reduction of ATP synthesis and mitochondrial membrane depolarisation [50]. FTC has also been shown to cause mitochondrial dysfunction when used together with TDF, the mechanisms of mitochondrial toxicity include a decrease in mitochondrial membrane potential, inhibition of OXPHOS complex I and complex iv enzymes, decrease in oxygen consumption, and increased production of mitochondrial ROS [58]. A previous study showed that TDF caused a significant decrease of ATP in mice kidney and a decrease in succinate dehydrogenase activity, which is also an indication of the loss of inner mitochondrial membrane integrity. Moreover, TDF accumulation within proximal renal tubules led to mitochondrial injury and depletion [59,60]. Another study showed that long-term treatment with HAART causes mitochondrial dysfunction in HIV patients [61].

EFV, the most popular NNRTI, has been associated with metabolic disorders, hepatic toxicity and neurotoxicity [62,63]. EFV effects on the mitochondria include a decrease in mitochondrial membrane potential, inhibition of OXPHOS complex I enzymes, decrease in oxygen consumption, decrease in ATP production and increased production of mitochondrial ROS [64,65,66]. Dolutegravir (DTG) an important INSTI class drug alters mitochondrial function by decreasing ATP synthesis, depolarising the mitochondrial membrane, and has the potential to alter immunometabolism [50]. Another HAART toxicity mechanism is through an induction of oxidative stress [49].

Oxidative stress, a state of imbalance between oxidants production and antioxidants, and mitochondrial impairment result from xenobiotic metabolism and accompany one another [67]. Disruptions to mitochondrial function increase the production of ROS, mostly superoxide, through impaired OXPHOS [68]. Increased free radical production, over a period of time, depletes the antioxidant defense response, eventually resulting in oxidative damage to macromolecules including DNA, protein and lipid membranes [69]. NRTI, NNRTI and INTSI of HAART are linked with increased levels of oxidative stres and depletion of antioxidants in HIV-infected individuals (Figure 1).

HAART (TDF,FTC,DTG) treatment to primary rat microglia increased ROS levels [70]. TDF also significantly increased ROS production, depleted antioxidant GSH and the mitochondrial superoxide dismutase (MnSOD) [71]. HAART (3TC and DTG in combination with Abacavir) have been reported to induce liver toxicity through upregulation of ROS [72,73]. 3TC and FTC induced hepatotoxicity by triggering oxidative stress and depletion of the antioxidants GSH and SOD1 while also increasing the expression of ALT [74]. EFV-treated SweAPP N2a neurons displayed enhanced release of ROS [75]. Hamed, Aremu [76] and Ikekpeazu, Orji [61] showed that GSH and GPx levels were significantly reduced in rats subjected to HAART (TDF, 3TC and EFV) Ikekpeazu, Orji [61] further showed that HAART increased levels of MDA, which is the biomarker for oxidative stress and a by-product of lipid peroxidation. HAART-induced oxidative stress has been demonstrated to interfere with the mitochondrial function leading to reduction in GSH content [77,78]. Prolonged oxidative stress is reported to trigger inflammation, which is exacerbated by HAART usage.

## 3. HAART-Induced Chronic Inflammation and Insulin Resistance

HAART reduces systemic inflammation and immune activation, but not to levels synchronous with HIV-uninfected populations. Furthermore, over a prolonged period, HAART induces inflammation. With effective viral replication suppression by HAART, there is still a heightened pro-inflammatory condition in treated people compared to non-HAART consuming individuals. This develops to chronic and systemic inflammation, which, over time, promote pathophysiological metabolic complications [79,80,81].

A chronic inflammatory state is based on evidence of increased levels of various pro-inflammatory cytokines, including tumor necrosis factor alpha (TNF-α) [82,83,84], interleukin 1 beta (IL-1β), interleukin 6 (IL-6) [80,83], and biomarkers of inflammation such as nuclear factor kappa B (NF-κB) and C-reactive protein (CRP) [85]. The stimulation and release of pro-inflammatory mediators from one site promotes inflammation and usually ends up interfering and affecting other tissues, thereby amplifying the chronic inflammatory state, impairment of the cellular pathologies, and eventually tissue dysfunction/damage [86].

A recent study reported that HAART (TDF, FTC, and DTG) increased the mRNA levels of IL-1β, IL-6, and TNF-α in rats [70], as TDF modulated mitochondrial biogenesis and triggered inflammatory pathways. A recent study showed that TDF induced pro-inflammatory cytokines TNF-α and IL-1β in mice [87]. Ramamoorthy, Abraham [88] showed that the activation of NF-kB and its downstream pro-inflammatory target genes, inducible nitric oxide synthase (iNOS), cyclooxygenase-2 (COX-2), and TNF-α, may play a critical role in the pathophysiology of TDF-induced renal damage in rats.

**Figure 2:**
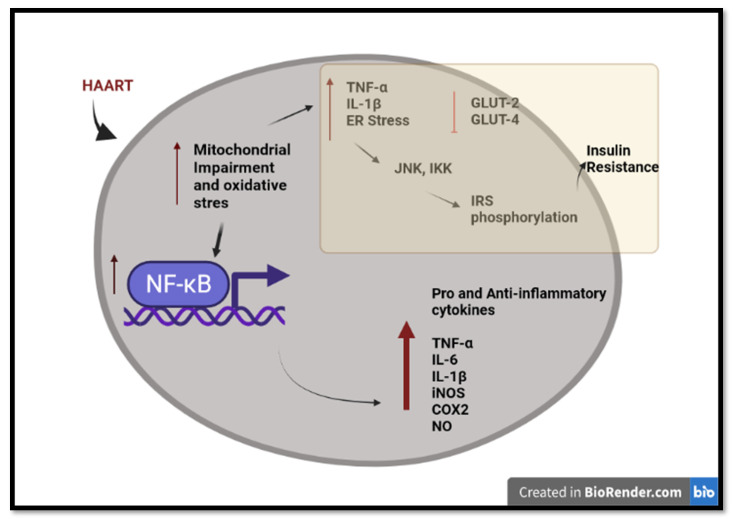
An overview of long-term HAART-induced inflammation and insulin resistance in HIV-infected individuals. HAART induced pro-inflammatory and anti-inflammatory cytokines through mitochondrial impairment, oxidative stress, and activation of NF-κB. HAART triggers insulin resistance via IRS phosphorylation and the inhibition of the glucose transporter. Created with BioRender.com (access date: 2 June 2022).

Hamed et al. (2021) reported that HAART (TDF, 3TC, and EFV) increased nitrite oxide (NO), a signaling molecule that plays a key role in the pathogenesis of inflammation. They also revealed that hepatic and renal membrane permeability, as well as caspase 3-dependent apoptosis, may be due to the stimulation of NF-kB and the enhancement of iNOS, essential factors of NO production. Moreover, it was reported that the oxidative stress induced by HAART may have triggered the inflammation (Figure 2). Oxidative stress can activate a variety of transcription factors, which lead to the differential expression of some of the genes involved in the inflammatory pathways [89,90]. Inflammation triggered by oxidative stress is the cause of many chronic diseases [91]. Chronic inflammation may cause pathophysiological complications such as insulin resistance [92,93,94].

Increased TNF-α levels affect the insulin receptor substrate (IRS) proteins, leading to insulin resistance [95]. TNF-α induces activation of serine kinases such as cJun N-terminal kinase (JNK) and the two-kinase complex (IKKalpha and IKKbeta) (IKK), which phosphorylates IRS-1. The increased concentration of phosphorylated IRS-1 inhibits the insulin receptor, thus causing insulin resistance [96]. Lastly, there is compelling evidence that HAART inhibits insulin-stimulated glucose disposal via the blockade of glucose uptake by glucose transporter isoform 4 (GLUT 4) and glucose transporter isoform 2 (GLUT 2). This leads to insulin resistance and impaired β-cell function via down-regulation of the insulin receptors [97,98]. EFV has been shown to increase blood glucose levels to a greater degree, while DTG triggers the development of insulin resistance in human adipocytes [99].

The primary goal of HAART is to suppress HIV replication, thus allowing for immune reconstitution and subsequent longevity in HIV-infected individuals. The safety of these drugs is of paramount importance and should continuously be evaluated to achieve optimum adherence and the benefit of the therapy, while maintaining its efficacy. As a supplement, the application of adjuvants can benefit the HIV-infected population. Alternatively, the use of medicinal plants that may synchronously function with HAART and hence may minimize the toxic effects of HAART. Medicinal plants are one of the most important sources of novel nutritionally and pharmacologically active compounds, and have a well-documented history in the prevention and treatment of various diseases [100]. They contain many bioactive compounds that act to minimize oxidative stress and inflammation [101]. One such plant is *Moringa oleifera* (MO).

## 4. *Moringa oleifera* (MO) as a Supplementary Medicine

MO is a medicinal plant of the *Moringaceae* family that was originally found in India and is now globally cultivated, including in SA [102]. Several studies have demonstrated the beneficial effects of MO in humans [20,102,103]. Different parts of the plant, such as the bark, leaves, seeds, flowers, roots, and immature pods, contain many important phytoconstituents [104]. The extracts of different parts of MO offer a high level of safety without any adverse effects to humans.

The leaves of MO are rich in minerals such as calcium, potassium, zinc, magnesium, iron, and copper, as well as vitamins (A, B, C, D, and E) [105,106]. MO leaves also contain phytochemicals such as tannins, sterols, flavonoids, saponins, alkaloids, terpenoids, anthraquinones, and reducing sugars, as well as anti-oxidative and anti-inflammatory agents such as glucosinolate, isothiocyanates, glycosides and glycerol-1-9 octadecanoate [22,105,107,108]. MO has been identified as an alternative protein source that can meet the regular demands of malnourished people [109] as it contains various types of amino acids. Essential amino acids such as methionine, cystine, tryptophan, lysine, caline, threonine, and isoleucine were found to be present in MO leaf extracts [20,23,36].

MO leaves also have a low calorific value and can be used in the diet of obese individuals. Many studies, both in vitro and in vivo, have confirmed the pharmacological properties of MO leaves (Leone et al., 2015)

### 4.1. Anti-Oxidant Properties of MO

The anti-oxidant properties of MO have been well documented [26,110,111]. MO is known as a free radical scavenger and extracts from the leaves exhibit a strong antioxidant activity against free radicals and prevent oxidative damage due to the enrichment of polyphenols [112,113]. MO leaves contain chlorogenic acid, rutin, quercetin glucoside, and kaempferol rhamnoglucoside [114,115].

MO was shown to restore glutathione (GSH), and increase the expression and activity of the glutathione-S transferase (GST), glutathione peroxidase (GPx), and glutathione reductase (GR) [111,116]. Increased GST activity leads to a larger detoxification of molecules through their conjugation with GSH. The synthesis of GSH depends mainly on the activity of gamma-glutamyl cysteine ligase (γ-GCL), which catalyzes the binding of glutamate to cysteine, the limiting step in the synthesis of GSH. MO induces the synthesis of the enzymes responsible for regenerating GSH levels, such as GR and γ-GCL [111,117]. This protective effect may be related to a variety of phytochemicals such as ascorbic acid and phenols (catechin, epicatechin, ferulic acid, ellagic acid, and myricetin) through scavenging free radicals [118,119]. Briefly, GSH, a potent endogenous antioxidant, scavenges electrophilic and oxidant species either in a direct way or through enzymatic catalysis: (i) it directly quenches reactive hydroxyl radicals, other oxygen-centered radicals, and radical centers on biomolecules. (ii) GSH is the co-substrate of GPx, permitting a reduction in peroxides (hydrogen and lipid peroxides) and producing GSSG. In turn, GSSG is reduced to 2 GSH via reduced nicotinamide adenine dinucleotide phosphate (NADPH) reducing the equivalents and glutathione disulphide reductase catalysis. Xenobiotic metabolites are conjugated with GSH through activation by GSTs [120,121,122].

The secondary antioxidant response includes many of the downstream genes responsible for regulating oxidative stress, and is regulated by the nuclear factor erythroid 2–related factor 2 (NRF2) [123]. NRF2 is normally maintained in the cytoplasm through interaction with the cytosolic repressor protein Keap1, an adaptor component of the Cullin 3-based ubiquitin E3 (Cul-E3) ligase complex, which promotes the ubiquitination and proteasomal degradation of NRF2. Exposure to both endogenous and exogenous molecules such as ROS lead to the dissociation of NRF2 from Keap1; NRF2 then translocates to the nucleus. In the nucleus, NRF2 heterodimerizes with small Maf proteins, making it bind to the *cis*-regulatory, antioxidant response element (ARE) located in the promoter region of NRF2 target genes, thereby activating their transcription. Target genes include hemeoxygenase-1(HO-1), superoxide dismutase (SOD), catalase (CAT), and NAD(P)H quinone dehydrogenase 1 (NQO1) [123,124].

MO upregulate NRF2 and attenuate oxidative stress; specifically, isothiocyanate from MO leaves have been shown to directly upregulate the NRF2 pathway [117,125]. Previous studies have shown that MO leaf extracts protect titanium dioxide nanoparticles and methotrexate-induced toxicity via upregulation of NRF2/HO-1 signaling and the amelioration of oxidative stress [126,127]. In other studies, MO drastically reduced lipid peroxides and increased GSH concentrations, along with a decrease in the activities of SOD and CAT [128].

### 4.2. Antiinflammatory Properties of MO

Chronic inflammation is involved in a number of disorders and is characterized by the continuous expression of pro-inflammatory factors and long-lasting tissue damage. MO possess properties that act against chronic inflammation and its associated disorders.

NF-κB is a transcription factor that is essential for inflammatory response. The NF-κB signaling pathway plays a role in the pathogenesis of liver injury caused by various agents [129,130,131]. IκB molecules sequester NF-κB in the cytosol of resting cells. Upon inflammation, IKK phosphorylation of IκB molecules promotes their degradation and releases NF-κB, which translocates to the nucleus to promote the transcription of target genes. NF-κB target genes include iNOS, TNFα, IL-6, IL-1β, and COX-2, which further mediate and propagate inflammation [132,133,134].

MO has been reported to decrease the production of TNF-α, IL-6, and IL-8 and the expression of *RelA,* a gene in (NF-κB) p65 signaling, during inflammation [135,136]. Previous studies have documented that MO can selectively inhibit the production of iNOS and COX-2, and significantly inhibit the secretion of NO and other inflammatory markers—including TNF-α, IL-6, and IL-1β in RAW264.7 cells and in human macrophages [30,136,137].

The isothiocyanate glycosides from MO leaves were shown to inhibit the expression of COX-2 and iNOS at both the protein and mRNA levels through inhibiting the major upstream signaling pathways via mitogen-activated protein kinases (MAPKs) and NF-κB [138,139]. Furthermore, Jaja-Chimedza, Graf [140] showed that MO leaves down-regulated the NF-κB pathway by decreasing the expression of IκBα, p-IκBα, and p65 proteins. MO also ameliorates inflammation by upregulating NRF2 [125,141]. Minaiyan, Asghari [142] showed potent inflammation attenuating actions of hydroalcoholic extracts of MO against inflammatory bowel ailments through diminishing the activities of IL6 and IL4, as well as TNFα, in rats.

Attenuating insulin resistance, MO increases the physiological activities of GLUT-2 and GLUT-4 in the plasma membrane, promoting the translocation of extracellular glucose into cells to increase the glucose consumption in cells [143,144]. Specifically, quercetin of MO leaf extracts has also been shown to activate AMPK, to increase glucose uptake through the stimulation of GLUT4 in the skeletal muscle, and to decrease the production of glucose through the downregulation of phosphoenolpyruvate carboxykinase (PEPCK) and glucose-6-phosphatase (G6Pase) (key enzymes involved in gluconeogenesis) in the liver [145]. Vargas-Sánchez, Garay-Jaramillo [146] stated that the flavonoid kaempferol has been shown to improve glycolysis, glucose uptake, glycogen synthesis, AMPK activity, and GLUT-4 expression.

## 5. HAART-MO

In vitro and in vivo studies have shown the toxicity of HAART [50,70,78,79,89,109]. Some clinical studies have shown the toxicity of HAART on the kidney, where TDF was associated with renal tubular dysfunction in HIV patients on HAART [57]. Patil, Ona [147] reported an acute liver toxicity of HAART in a male patient. The prevalence of metabolic complications in HIV individuals consuming HAART have been reported [148,149,150]. In vitro and in vivo studies have outlined the therapeutic effects of MO bioactive compounds at a molecular and cellular level, and a few clinical studies on the MO therapeutic effects have been conducted [151,152,153]. The anti-asthmatic activities of MO in patients have been reported [154], and Taweerutchana, Lumlerdkij [151] reported that MO leaf reduced blood pressure in diabetic patients.

Intriguingly, MO therapeutic effects have been shown for the immune status of HIV-infected people on ART as well. A study on adult patients revealed that MO leaf powder supplementation improved the body mass index and immune response in HIV patients on ART [155]. MO leaf supplementation was shown to be associated with increased CD4 cell counts of PLHIV on ART [156]. Ogunlade, Jeje [157] showed that MO leaf extract restored semen quality, hormonal profile, and testicular morphology against HAART-induced toxicity in adult male Wistar rats.

Although the clinical application of MO is not comprehensive enough yet, we can at least conclude from the previous demonstrated studies that MO could be used as a therapy and a supplement, or as an adjuvant in the treatment of metabolic disease complications. Therefore, considering the molecular and cellular toxicities induced by HAART, and the therapeutic benefits of MO, the pathophysiological complications experienced by HIV-infected people consuming HAART may be attenuated.

Taken together, metabolic disorders in HAART consuming individuals develop and progress primarily as a result of impairments in the metabolic pathways. The approach for improving and advancing HAART may be exploring and targeting cellular pathways, such oxidative stress and inflammation. Medicinal plants are either used as crude or purified extracts. The medicinal plant MO is therapeutic and safer as it is rich in phytochemicals and displays potent antioxidant and anti-inflammatory activities.

## 6. Conclusions

Long-term HAART consumption leads to toxicity, mainly through oxidative stress and inflammation, which become clinically visible as MetS. MO leaves possess bioactive compounds that have antioxidants and anti-inflammatory properties. Therefore, MO leaves could be a great supplementary medicine to ameliorate HAART-induced toxicity. In addition, specific MO bioactive compounds such as quercetin could be extracted and used as an optimized adjuvant for improving HAART. MO agents could be a source of easily accessible and affordable therapies against HAART toxicities in the future.

## Data Availability

Not applicable.
